# *In vitro* characterization of biofilm produced by *Fusarium oxysporum*, an onychomycosis agent^☆^

**DOI:** 10.1016/j.abd.2024.11.005

**Published:** 2025-04-21

**Authors:** Isabella Letícia Esteves Barros, Flávia Franco Veiga, Terezinha Inez Estivalet Svidzinski

**Affiliations:** a Postgraduate Program in Health Sciences, Universidade Estadual de Maringá, Maringá, PR, Brazil; b Medical Mycology Laboratory, Department of Clinical Analysis and Biomedicine, Universidade Estadual de Maringá, Maringá, PR, Brazil

Dear Editor,

Onychomycosis caused by Non-Dermatophyte fungi (NDMs), such as *Fusarium* spp., is more prevalent than previously thought, especially in warmer climates.^1^ Furthermore, onychomycosis has currently been attributed to fungi organizing themselves into a biofilm form.^2^ Biofilm is a complex microbial community, highly adhered to the nail and surrounded by a matrix that provides protection and antifungal resistance.^2^, ^3^

The research group has been studying the genus *Fusarium* spp. as an agent of onychomycosis in immunocompetent hosts. The authors reported its high prevalence in the studied region, established clinical and laboratory criteria for this genus as a causal agent of onychomycosis, and determined the susceptibility profile to the systemic antifungals most commonly used in Brazil.^4^ Later, the authors proved that *Fusarium* spp. uses nail keratin as a single source of nutrients^5^ and began studies on the etiopathogenesis of fusarial onychomycosis based on an *ex vivo* model using sterile human nail fragments.^3^

More recently the authors reported, for the first time, that *Fusarium oxysporum* is able to form biofilm on the human nail as the only nutritional source.^6^, ^7^ Also, the authors describe the volatile organic molecule 2-Ethyl-1-Hexanol (2 EH) as a quorum-sensing component capable of modulating such biofilm.^8^ These findings were relevant to confirm the etiopathogenesis of fusarial onychomycosis. However, it did not reveal the proper characteristics of the biofilm formed under nutritional support. Thus, the current study aimed to characterize the *in vitro* biofilm formation of *F. oxysporum*, evaluating its natural ability, during 7-days with controlled nutrient availability.

This study was conducted with *F. oxysporum* CMRP2925 isolated from a previously described onychomycosis case.^4^ The isolate was reactivated to confirm its purity and identification, before assays, and was cultured on Sabouraud Dextrose Agar (SDA; DifcoTM, MI, USA) for 7-days at 25 °C. Biofilms were prepared according to Galletti et al.,^9^ with some modifications. A suspension containing 1 × 10^7^ conidia mL^−1^ was prepared in RPMI Medium 1640 (Gibco, NY, USA), with L-glutamine, sodium bicarbonate, 0.165 M 3-(N-morpholino) propanesulfonic acid (pH 7.2), and 2% glucose. This suspension was placed into 96-well flat-bottomed microtitration plates and incubated at 35 °C in a shaker at 110 rev min^−1^, for 7-days. Every 24 h, the culture medium was renewed by removing 100 μL of old broth and adding the same volume of fresh RPMI. During the seven days, biofilms were evaluated under different aspects, as previously described.^6^, ^9^ Briefly, cell viability was assessed by counting Colony Forming Units (CFU), quantification of total biomass by Crystal Violet, metabolic activity by the reduction assay of tetrazolium salt, 2,3-(2-methoxy-4-nitro-5-sulphophenyl)-5-([phenylamino]carbonyl)-2H Tetrazolium hydroxide (XTT), characterization of the Extracellular Matrix (ECM), and visualization of biofilm structure by Scanning Electron Microscopy (SEM).

Overall, the biofilm’s characteristics were similar, drawing a parallel between greater nutritional support with RPMI and nails only.^6^ In both situations, it was possible to show that the third and fourth days were critical. In rich medium and neutral pH, the number of CFU remained constant at all times (Fig. 1A), since the first day, with an increase in metabolic activity on the third day, stabilizing from this moment. A similar profile was found for proteins and nucleic acids. Interestingly, polysaccharides were significantly consumed until the third day, continuing to decline gradually until the seventh day. On the other hand, using the nail as a nutritional source had observed an increase in CFU until the third day, a high metabolic activity only on the fourth day, while the increase in proteins and carbohydrates occurred late, corroborating the results of nucleic acids.^6^ These differences can be attributed to the availability of nutrients, with daily renewal of the RPMI medium (food at will), and the fact that in the nail (restricted food), the fungus itself needed a period of adaptation.

**Fig. 1:**
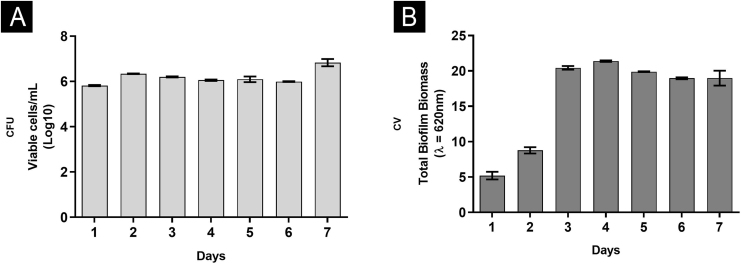
Characterization of biofilm produced by *F. oxysporum* on flat-bottomed polystyrene plates from one to seven days, in RPMI medium. (A) Number of viable cells by counting Colony-Forming Units (CFU); (B) Total biomass by Crystal Violet staining (CV).

In addition, a progressive increase in the amount of biomass was observed (Fig. 1B) between the first and third day, followed by stability, which seems to be associated with the consumption of polysaccharides (Fig. 2), a fundamental component during the development and maintenance of the biofilm. Taking into account that the CFU remained stable, this increase was attributed to the large production of ECM, in a rich environment. The increase in metabolic activity (Fig. 2) is reflected in protein and nucleic acid synthesis, mainly eRNA. Protein production appears to be crucial in the process of biofilm formation and support of the entire system.^6^, ^10^ This idea is reinforced by the high levels of XTT and eDNA, from the fourth day onwards, since this nucleic acid is needed in activities that demand cellular energy, such as the construction of a mature biofilm.^10^ The biofilm structure begins by forming a cell monolayer and within seven days the highly complex three-dimensional structure was observed (Fig. 3A and B).

**Fig. 2:**
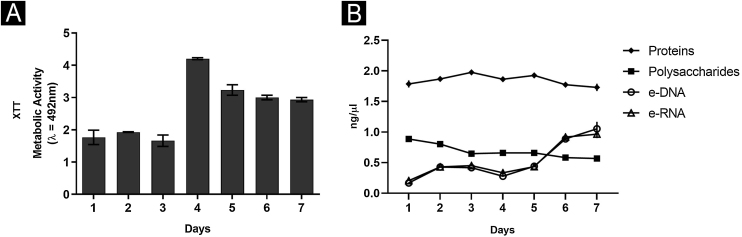
Characterization of biofilm produced by *F. oxysporum* on flat-bottomed polystyrene plates from one to seven days, in RPMI medium. (A) Metabolic activity by XTT reduction; (B) Quantification of Extracellular Matrix (ECM): proteins, polysaccharides, extracellular DNA and RNA.

**Fig. 3:**
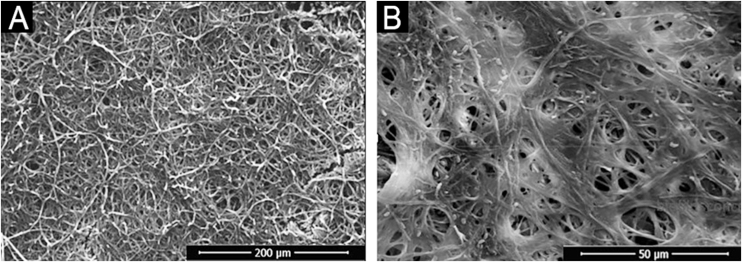
SEM illustration of four-day *F. oxysporum* biofilm structure. (A) Highlighting the structural organization of the biofilm, the intertwining and compaction of the hyphae (500×); (B) Higher magnification, showing the extracellular matrix produced (2000×).

Thus, it is logical to assume that the ability of *F. oxysporum* to produce biofilm is intrinsic. The nutritional support seems just to facilitate the first hours of its formation, in addition to favoring the production of a greater amount of biochemical components associated with the maturation and stability of the biofilm. Therefore, the experience draws attention to the natural abilities of this fungus, previously restricted to the environmental, agricultural, and animal interest. Probably, the natural selection induced by pesticides facilitated its adaptation to human tissues. The authors were able to show its virulence potential (here the efficiency in producing biofilm) is maintained regardless of the addition of nutritional support. This behavior therefore allows us to consider the genus *Fusarium* spp. as a primary pathogen of interest in dermatology.

## Financial support

This work received partial financial support from Coordenação de Aperfeiçoamento de Pessoal de Nível Superior ‒ Brazil (CAPES) (Finance Code 001, through a scholarship awarded to ILEB); Conselho Nacional de Desenvolvimento Científico e Tecnológico ‒ Brasil (Process number 3128262023-0 given to TIES) and INCT-CERBC, 2022, Process number 406645/2022-1.

## Authors’ contributions

Terezinha Inez Estivalet Svidzinski: Came up with the research idea, planned the experiments, and proofread them.

Isabella Letícia Esteves Barros: Came up with the research idea and planned the experiments; Performed the experiments and analyzed the data; Wrote the manuscript.

Flávia Franco Veiga: Performed the experiments and analyzed the data.

## Conflicts of interest

None declared.
